# Comparison of multimodal active learning and single-modality procedural simulation for central venous catheter insertion for incoming residents in anesthesiology: a prospective and randomized study

**DOI:** 10.1186/s12909-022-03437-0

**Published:** 2022-05-11

**Authors:** Antonia Blanie, Cécile Shoaleh, Fabien Marquion, Dan Benhamou

**Affiliations:** 1 Centre de simulation LabForSIMS, Faculté de médecine Université Paris Saclay, 94275 Le Kremlin-Bicêtre, France; 2 Département d’Anesthésie-Réanimation, CHU Bicêtre, 94275 Le Kremlin Bicêtre, France; 3 CIAMS, Univ. Paris-Saclay, Université Paris-Saclay, 91405 Orsay Cedex, France; 4 Université d’Orléans, 45067 Orléans, France

**Keywords:** Active learning, Central venous catheter, Flipped classroom, Simulation, Peyton’s 4-step, Kirkpatrick

## Abstract

**Background:**

Active learning methods, including low-fidelity simulation, are useful but the incremental learning effect of each method is however limited. We designed this study to assess if combining flipped classroom and the modified Peyton’s « 4-steps» method during procedural simulation (intervention group [IG]) would provide better learning results than simulation alone (control group [CG]) in the context of central venous catheter insertion training.

**Methods:**

This prospective, single-center, and randomized study took place in 2017 in a single simulation center. All first year Anesthesiology residents of Ile de France area at the start of their residency were randomly included either in the IG or CG during a seminar aimed at providing initial procedural skills with low-fidelity simulation. A composite learning score which included knowledge MCQ and a questionnaire assessing satisfaction and value of the training session was recorded after training (primary outcome, /100). A randomized sub-group of learners of each group were video-recorded during central venous catheter insertion at the end of training and their skills were evaluated with validated tools, including a specific checklist and a global rating scale (GRS).

**Results:**

Among 89 anesthesiology residents, 48 and 41 learners were randomized in the intervention and control groups respectively. Of the IG residents, 40/48 (83%) had read the learning material prior to the session. There was no significant difference regarding the composite outcome ([IG]= 81.1 vs [CG] = 80.5 /100 (*p* = 0.68))*.* Results of the post-session MCQ knowledge questionnaire were also non-significantly different. Residents were similarly satisfied and described a significant improvement of their knowledge and skills after training. Learners highly valued the training session as a mean to improve their future practice. No significant differences regarding checklist and GRS scores were observed.

**Conclusions:**

A multimodal active learning strategy of procedural learning did not provide better learning outcomes when compared to a traditional simulation method. In both groups, satisfaction was high and perception of the acquired theoretical and practical knowledge was improved after training.

**Supplementary Information:**

The online version contains supplementary material available at 10.1186/s12909-022-03437-0.

## Background

Learning technical procedures is extremely important for healthcare professions who perform complex invasive procedures on patients.. Launched in 2017 in France, an-in depth reform modifies the training strategy for Anesthesiology residency [1] and highlights “active” teaching techniques, competency-based education and the use of entrustable professional activities. In addition, this type of learning should be introduced at the start of residency, even before residents are in first contact with patients. Training for the placement of a central venous catheter (CVC) has been highlighted as an essential procedure .

While there is unanimous agreement on the principle of using active learning, the best method is still being discussed and these educational methods have a moderate impact on learning outcomes in the short term. Simulation is a well-accepted method of active learning and its use has spread worldwide [2]. The effect size of learning improvement is however moderate, superior to no-training but not significantly better than traditional methods [3] for its effects on patients’ outcomes [4]. In the context of CVC insertion, the meta-analysis of Madenci et al., has shown that a significantly larger proportion of those trained with low-fidelity simulation successfully placed CVCs and required fewer attempts to CVC insertion compared to traditional group although no significant difference in the rate of adverse events between the groups were observed [5]. Since 2014, several studies have confirmed these results although providing low-grade conclusions because of study heterogeneity or non-randomized designs [2, 6, 7]. Indeed, the gain expected from a teaching method is generally said to be satisfactory if in the order of one standard deviation of the value obtained with another method [7]. Some authors have also used a combination of methods, hoping to reinforce the educational effect [8, 9].

The aim of the study was to assess if multimodal active learning (combining flipped classroom and the modified Peyton’s « 4-steps» method during procedural simulation, intervention group [IG]) would provide better learning results than simulation alone (control group [CG]) in the context of central venous catheter (CVC) insertion training.

## Methods

### Study design

**Fig. 1:**
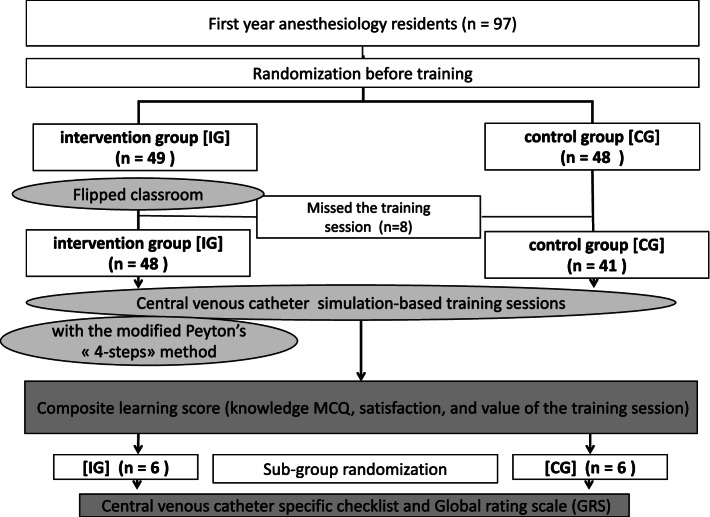
Study flow chart

This study was a prospective, single-center, randomized, open label study (Fig. 1).

After written consent, all incoming first year Anesthesiology residents of the Ile de France area in the 2017–2018 academic year who were attending their mandatory simulation training were included.

The boot camp was organized during the first days of residency and lasted a total of three days and included theoretical and low-fidelity simulation sessions (2017 November). The part of the training program dedicated to teaching the CVC placement procedure was performed at the LabForSIMS simulation center (Kremlin Bicêtre Medical School of Paris Saclay University). Its content and educational objectives had been prepared by anesthesiology experts with a large experience in simulation. During sessions, the overall number of instructors was one for 5 trainees.

The duration of the planned CVC workshop was the same for both groups (two hours) and consisted of three different stations. The first 30-minute station was dedicated to patient preparation and relationship, antiseptic preparation, and introduction to the principles of interprofessional collaboration. The second 45-minute station was dedicated to the general principles of using ultrasound for needle puncture (Ultrasound Training Block Model, CAE Healthcare, USA) The third 45-minute station was based on ultrasound guidance for internal jugular CVC placement (Seldinger technique and out-of-plane approach) performed on a task trainer (Ultrasound Catheter Insertion low fidelity Simulator, Kyoto Kagaku®, Ref: KKM93UB, distributed by Medicalem, France) (ultrasound machine, Mindray®, model TE7) (CVC, Arrow®, Ref: CV-04301).

Residents were randomly divided into two groups who were trained to CVC placement prior or after a training session on cardiopulmonary resuscitation. Because of this distribution, a total of six groups were constituted and a randomization list was made to define the order of sessions on each day (by random Excel).

In the control group (CG), each station began with a 15 min slide presentation, to describe the main principles of the theme studied. During the third station in which the complete procedure was performed, the instructor described the CVC insertion procedure on a task trainer while performing the procedure. Then the learners performed the procedure according to the time available (but at least once per trainee), with immediate correction by the instructor and answering questions as the procedure progressed.

In the intervention group (IG), the students had received the theoretical teaching documents by individualized e-mail explaining the need to read the documents sent in advance of the session (flipped classroom model [10]) and explaining that all details will not be presented during the session, so as to keep more time for active learning. Documents were sent 1 week before the session and contained three reviews (i.e. one describing French guidelines for antiseptic preparation of a healthy skin [11], a review describing the important steps of ultrasound-guided CVC placement [12], a CVC placement checklist example [13] and a link to a video showing best practice CVC placement [14]. The workshop started by questioning learners on their understanding of the documents read beforehand, followed by a reminder of the indications of the use of CVC and the key messages of the documents. Then, procedural training followed the four Peyton’s steps which involved demonstration, deconstruction in micro-tasks, learner performance and observation by the instructor and peers [15]. Finally, the trainer summarized essential points observed (positive) and errors to be avoided.

### Evaluation of learners

The training evaluation was conducted according to Kirkpatrick’s classification [16].

At the end of the simulation session, all learners completed a questionnaire to define their general characteristics and a knowledge questionnaire (20 Multiple Choice Questions [MCQ]). This questionnaire had been prepared by the group of expert trainers,was adjusted to the content of the stations and validated by the authors. The questionnaire also addressed self-assessment of satisfaction, perception of skills acquisition (theoretical and practical), and of the value of the training to improve their future practice (Kirkpatrick levels 1 and 2, subjective assessment). Questions were presented in the form of a Likert scale from 0 (no effect) to 10 (excellent).

A composite learning score (primary outcome) was composed to provide an overview of learning after training and combined perception of acquired theoretical (/10) and practical (/10) skills for CVC placement after the session, knowledge MCQs (total score /20), satisfaction (/10) and usefulness of the training session for their future professional practice (/10). The final composite outcome score could thus have a maximum value of 60, which was transformed to reach 100 to facilitate analysis.

In each group, at the end of each session, a sub-group of six learners were drawn at random and after signing authorization allowing for video recording, they proceeded to perform the jugular CVC procedure on the same task trainer (secondary outcome). During the procedure, the learner was stand-alone, but an instructor played the role of a nurse assisting the physician. Videos were analyzed a posteriori by two investigators separately who were unaware of the randomization list. Each video assessed the impact of the session on the acquisition of technical skills using two evaluation methods (Additional file 1): 1) a specific checklist for CVC placement modified from Hartman et al. [17] after exclusion of 11 of the 30 items since some items of the checklist could not be studied (i.e. obtaining the patient’s consent, choice of site, etc.). Analysis was therefore based on 19 items and evaluated the important technical steps for the CVC insertion procedure; 2) a Global Rating Scale (GRS) for a total of 30 points (five points per item). In the event of major disagreement on scoring after comparing results, the two assessors had to review the recordings together again to find an agreement on scoring for the given learner.

### Statistical analysis

The primary outcome was the composite outcome aimed at discerning whether a better learning effect could be seen with the multimodal training strategy.

Secondary outcome criteria included results obtained with the GRS and the checklist scores, and separate analysis of each component of the composite score.

We assumed that the mean value of the composite score would be 80/100 in the CG with a standard deviation of 10/100 and that a progression in the intervention group of at least 1 standard deviation is significant and usually accepted in studies carried out in the field of education [15], (expected increase to 90/100). Considering an alpha risk at 0.05 and a power (1-beta) at 0.9 using a one-tailed test, 38 residents per group had to be included in each group to observe a significant difference (https://marne.u707.jussieu.fr/biostatgv/?module=etudes/sujets#).

After checking for normal distribution, results were presented as mean and standard deviation or as percentages. Comparisons were made using Student’s t-test or ANOVA for repeated measures for continuous and parametric data. For non-parametric data and percentages, the comparisons used either a Wilcoxon test or a Chi-squared test. A *p*-value of less than 0.05 was considered significant. The software used was JMP® (SAS Institute, USA).

### Regulatory and ethical aspects

The protocol, the information and written consent forms and the form requesting authorization to use the videos for educational and research purposes were submitted to the Ethics Committee for Research (CERAR) of the French Society of Anesthesia and Intensive Care Medicine (SFAR), and approval has been obtained (IRB 00010254–2017 – 004).

## Results

Of the 97 incoming residents randomized, 89 participated in the boot camp (Table 1), 48 and 41 learners in the intervention and control groups respectively while 8 residents who missed the training session were excluded. There were no significant differences for age, gender, or clinical experience during undergraduate training between the two groups. In the IG group, all learners had received the training materials prior to the session. Learners’ adherence to the flipped classroom process class was 83% (40/48 reporting having read the materials received prior to the training). The non-readers were not excluded from the study.

**Table 1:** Characteristics of incoming first year anesthesiology residents participating in the study

	Intervention Group (***n*** = 48)	Control Group (***n*** = 41)	***P***
**Age** (years)	24.8 ± 1.2	24.8 ± 1.3	0.97
**Gender M/F** (n)	16/32	17/24	0.51
**Completion of an Intensive Care rotation during undergraduate training** (n, %)	38 (79)	32 (78)	0.58
**Completion of a Pre-hospital / Emergency rotation during undergraduate training** (n, %)	24 (50)	28 (68)	0.19
**Completion of an Anesthesiology rotation during undergraduate training** (n, %)	21 (44)	22 (54)	0.67

Results presented as mean ± standard deviation or n (%)

There were no significant differences regarding the composite outcome (Table 2). Theoretical knowledge scores (MCQ) measured after the session were not significantly different between the two groups. Learners’ perceptions of their theoretical and practical knowledge of CVC placement was assessed as being overall two-fold higher in each group after receiving training (*p* < 0.001 for all questions), but without any significant difference between groups (Table 2). Learners highly valued the training session as a mean to improve their future practice, but there were no significant differences between groups (Table 2).

**Table 2:** Analysis of theoretical knowledge and practical skills regarding CVC placement before and after the training session

Questionnaire items	Intervention Group (***n*** = 48)	Control Group (***n*** = 41)	***P***
Composite outcome score ^a^ ([A + B + C + D + E] × 100/60)	81.1 ± 7.1	80.5 ± 5.8	0.68
A - Learners’ perception of their theoretical skills for CVC placement *after* the session (0–10) ^a^	8.2 ± 1.2	8.2 ± 1.0	0.88
B - Learners’ perception of their practical skills for CVC placement *after* the session (0–10) ^a^	7.3 ± 1.3	7.5 ± 1.1	0.42
C - Knowledge MCQs, total score (out of 20)	15.5 ± 1.7	15.3 ± 2.4	0.69
D - Overall satisfaction with training (0–10)	8.9 ± 1,.1	8.7 ± 1.0	0.34
E - Usefulness of the training session on the future change of professional practice (0–10)	8.7 ± 1.8	8.6 ± 1.2	0.70
Learners’ perception of their practical knowledge of CVC placement *prior* to the session (0–10) ^b^	4.1 ± 3.1	3.5 ± 2.7	0.30
Learners’ perception of their theoretical knowledge of CVC placement *prior* to the session (0–10) ^b^	4.8 ± 2.7	4.0 ± 2.6	0.16
Learners’ perception of their CVC placement theoretical knowledge (difference between *after – before) (*%)	+ 3.5 ± 2.5	+ 4.2 ± 2.6	0.16
Learners’ perception of their CVC placement practical knowledge (difference between *after – before) (*%)	+ 3.3 ± 3.0	+ 4.1 ± 2.7	0.21

Results presented as mean ± standard deviation. CVC: central venous catheter

^a^Composite outcome score: out of 60 transformed of 100

^b^Learners’ perceptions of their theoretical and practical knowledge of CVC placement was felt to be overall two-fold higher in each group after training (*p* < 0.001 for all questions) but without significant differences between groups

Scores obtained with the checklist (intervention group: 17.2 ± 1.1 versus control group: 17.5 ± 1,3; *p* = 0.65) and the GRS (Table 3) were no significantly different between the two sub-groups.

**Table 3:** Results of the Global rating Scale (GRS) assessment grid [17]

GRS Item (0 to 5 points per item)	Intervention Group (***n*** = 6)	Control Group (***n*** = 6)	P
Knowledge of specific procedure	3.7 ± 0.9	3.6 ± 0.9	0.87
Knowledge of equipment	3.0 ± 0.9	3.1 ± 1.0	0.88
Flow of procedure	2.5 ± 0.7	2.8 ± 1.2	0.57
Time and motion	3.1 ± 0.7	2.8 ± 1.0	0.63
Instrument handling	3.2 ± 0.5	3.3 ± 0.8	0.83
Bimanual dexterity	3.8 ± 0.8	3.8 ± 0.5	1.0
**TOTAL (out of 30 points)**	19.2 ± 3.4	19.3 ± 4.6	0.94

Mean scores ± standard deviation

## Discussion

This study compared a single conventional procedural simulation training method to a multimodal combination of three active learning methods which included a flipped classroom strategy, a modified Peyton’s “ 4 - steps” method used during the procedural simulation session. Both methods were applied with the goal to train incoming Anesthesiology residents to place a CVC. Both groups were highly satisfied with the training method they received and believed that the training session would be useful for their future practice. By contrast, no difference was found between the two training methods for both subjective and objective measurements, including the composite learning outcome and procedural skills gained during the training session.

The implementation of a teaching system based on active learning, and especially with simulation based-education, requires more human and financial resources than traditional methods (low learner/trainer ratio, high-quality materials (high and low-fidelity simulators), cost of sophisticated computer programs such as those used in “serious games”). It is therefore important to have a factual benefit to encourage this change and obtain the necessary funding. In a review describing the value of active learning in various scientific topics, Freeman et al. [18] demonstrated that it reduces the failure rate of students and increases the transition rate to the next grade. Active learning motivates students in the learning process to take part in activities and/or discussion in the classroom, as opposed to passive learning which is usually done by listening to an expert. Active learning emphasizes the benefit of enhanced reflection and may include group work. However, this review did not include healthcare sciences in which the evidence is weaker. The systematic review and meta-analysis of Brydges et al. [3] shows that although simulation significantly improves satisfaction and subjective perception of gaining knowledge, simulation only improves medical care outcomes compared to no intervention. In support of this limited effect, note that in the review by Freeman et al. [18], the improvement in learner success rates was only about 15%. Experts in educational sciences are well aware that this limited effect is common to all learning methods, making it difficult to demonstrate their benefit and some of them even reject both the need to demonstrate an effect and the use of randomized designs for this purpose [19]. It should be emphasized that in the above-mentioned studies, the effect of a single active learning technique was evaluated. One possible mean to overcome the limited power of each active learning methods could be to combine several active learning methods in order to add (or hopefully potentiate) the benefit obtained by each method. Our study therefore wished to evaluate the pedagogical value of a combination of active learning methods.

The lack of benefit of our multimodal strategy observed in this study may be the result of a real lack of educational potency difference with a single-active learning. In fact, a testing effect might have played a role in the two groups as an additional learning method [20] but evaluation was not aimed at being summative but rather to assess the value of the training method.

As we have seen above, the pedagogical effect size of these new methods remains limited [4]. It is possible that most training programs using simulation are too time-limited and do not include enough time within each simulation session. However, even in programs in which the place of simulation is high, the benefit may be limited [21]. An alternative possibility is that simulation alone produces an effect potent enough to obscure the additional effect of any other active learning method added. The Peyton’s method is believed to be a better practical learning method by deconstructing the technical procedure [15] but other studies have not found any difference [22], again suggesting a limited educational effectiveness. The principle of the flipped classroom by freeing the teacher from teaching basic data, is also a method that requires an effort from the learner, who not only becomes more active in his (her) own learning but also learns to master the technological tools and creates stronger conditions for interaction during the teaching session. The well-known risk of learner buy-in was not seen in our study. Numerous studies and meta-analyses have however provided controversial results regarding its educational effect [10, 23].

We are therefore facing modern methods that increase students’ motivation but which ultimate pedagogical value remains uncertain. In the context of the present study, their addition did not give a more convincing result than their separate use. Our study showed an immediate improvement in learners’ perceptions of their technical knowledge and skills in both groups. Future studies should assess if the use of active healthcare education methods could improve knowledge retention and professional practice in the long-term through better adherence and motivation of students.

We believe that there is a need to review the educational capacity of the many methods of active learning in order to identify those which have a truly significant intrinsic potential. The idea of combining several strategies should be analyzed further, despite the inconclusive results observed here. The need to combine an even greater number of active approaches in order to obtain a significant pedagogical effect could also be explored.

Our results are also confirmed by many studies which have shown that active learning improves learners’ satisfaction and motivation as well as perception of a useful training [10, 24, 25]. By contrast we also included objective measurement (MCQ score) and assessed technical skills with well-accepted scoring systems. The literature review [26, 27] suggests that the combination of a GRS and a specific checklist is better than the use of either method alone to evaluate technical procedures. Video recording was also a great addition and increased the value of our measurements although we were only able to record a limited number of learners which reduces the power for this outcome. Retrospective analysis could be made in a quiet environment, separately by each of the two assessors who were blinded to the study group and reconciled their notes by reviewing together the video files when needed.

Many studies, one systematic review and one meta-analysis have shown the effectiveness of boot camps on the acquisition of skills in learners [28, 29]. For example, the prospective study by Bamford et al. [24] compared how novice surgical trainees felt before and after participating in a boot camp. At the end of the training program, they described an improved ability to manage patients with multiple trauma or organ failure. They had also improved essential technical and non-technical skills such as leadership, communication and vigilance. Examples of boot camp studies in Anesthesiology are however rare [30, 31].

The study has also significant limitations. First, we chose a composite outcome aimed at discerning whether a better learning effect could be seen with the multimodal training strategy. It is well known that identifying such an effect is difficult and many studies have failed although participants and assessors had a positive view of the method assessed. In addition, the learning change, as measured by a single outcome measure, is often small, leading education specialists to consider that an increase of one standard deviation would reflect a “significant” change. To overcome this difficulty, we decided to use a composite outcome [32] which was thought to better reflect the overall educational value of each of the compared techniques. It could be argued that the composite outcome has limited statistical significance since it combines subjective (questionnaires) and objective measurements (MCQ). We feel however that this is relevant to education research since all components of our composite outcome are parts of the overall desired outcome measured and are well associated with the primary objective of the study (i.e. assessment of the benefit of the training method). Because each measure has a different goal, this overcomes concerns about correlation between individual outcomes. Moreover, self-report measures cannot be accepted as the only outcome measure but the use of composite outcome allows to combine self-report questionnaires and objective measurements. In addition, mixed methods are useful in this setting [33]. Demonstrating positive effects on satisfaction or feeling of realism (Kirkpatrick 1 level of assessment) is debatable because they reflect only subjective outcomes and this might not be enough to implement a new educational technique such as simulation, given the potential drawbacks such as costs. MCQs were also used and are more objective measurements but when a procedural skill is assessed, they may not be the best outcome to assess performance if used alone. The absence of difference between the two educational strategies, as shown by the composite outcome, was confirmed by comparison of its individual components and by the direct assessment of procedural skills measured in a subset of learners.

Second, although acquisitions were evaluated by means of MCQ (knowledge), and the technical skills by means of a task trainer procedure, these methods of evaluation do not always correspond to the actual skills of the students, observed in clinical practice [34]. Moreover, for some shy students, being in a small group, being questioned or filmed can significantly increase their stress and compromise their skills.

Finally, despite the numerical imbalance between the two groups, the residents were evenly distributed with respect to their initial characteristics while a similar and adequate number of trainees were assessed for all outcomes. The prospective, controlled and randomized design of the study was robust. The boot camp program was part of the official curriculum with a well-defined outline. Therefore, despite the fact that this was a single center study, its results are likely reproducible and can be extrapolated to other training centers at the national level. Karpicke JD et al. [35] suggested the importance of carrying out tests in a repetitive way because repetition increases the retention of knowledge. However, because of time constraints, the learners in our study did not take the pre-test nor a long-term assessment. Our trainees’ previous experience was not significantly different between groups and the number of residents who had made a rotation in Anesthesiology or Intensive Care unit during their clerkship was not different. It is of note that medical students are not allowed to perform CVC placement.

Pretest is not necessary when a randomization has been conducted and the sample is sufficiently large as in our study [36]. Moreover, pretest is not learning neutral. It provides the participants with foreknowledge of the posttest, so is part of the intervention that we didn’t want in this study. Our trainees were assessed immediately after a single training session although long-term knowledge and skills decay is a well-known risk for many procedures including CVC placement [7]. Moreover, long-term analysis of the educational benefit of a training session depends on the subsequent career path of the resident and the number of the given procedure performed.

## Conclusion

Among the incoming Anesthesiology residents studied, no significant difference on acquisition of CVC placement technical skills was found at early assessment between a multimodal combination of active learning techniques and a single procedural simulation modality. The learners’ perception of their theoretical and practical skills for the procedure increased significantly after training. Further studies are needed to assess the impact of this increased motivation on long-term professional and patients’ outcomes.

## Supplementary Information

**Additional file 1.**

## Data Availability

The datasets generated during and/or analyzed during the current study are available in the figshare.com repository, [10.6084/m9.figshare.19174946.v1].

## Abbreviations

CVC: central venous catheter
IG: intervention group
CG: control group
