# Supplementary material for: Comparison of multimodal active learning and single-modality procedural simulation for central venous catheter insertion for incoming residents in anesthesiology: a prospective and randomized study
Source: BMC Med Educ. 2022 May 11;22:357. doi: 10.1186/s12909-022-03437-0 (PMC9092872; doi:10.1186/s12909-022-03437-0)
Supplement: Supplementary file 1 — Additional file 1. [file 12909_2022_3437_MOESM1_ESM.docx]

## Modified specific checklist template for the insertion of a central venous catheter (18).

|  | Procedural stain | Done | Not done |
| --- | --- | --- | --- |
| 1 | Can describe the indication of the procedure |  |  |
| 2 | Appropriate antiseptic detersion with CHX-alcohol |  |  |
| 3 | Washing hands after putting on mask and cap |  |  |
| 4 | Wears gown and gloves |  |  |
| 5 | Covers the patient with wide fields |  |  |
| 6 | Covers the ultrasound probe with the sterile sheath |  |  |
| 7 | Anaesthetizes the skin and the entire puncture area |  |  |
| 8 | Re-identifies the vein with the ultrasound, checks for compressibility and locates the carotid artery. |  |  |
| 9 | Under ultrasound control, slowly advance the needle by sucking in |  |  |
| 10 | When he get into the blood vessel, make sure it's not the artery. |  |  |
| 11 | Hold the needle in place and insert the metal leader (remove the syringe or insert the leader into the Raulerson syringe). |  |  |
| 12 | Moves the fence forward without forcing and checks that it can be moved in both directions without hindrance. |  |  |
| 13 | Remove the needle by checking the metal guide. |  |  |
| 14 | Inserts the dilator with a rotating movement and then removes it. |  |  |
| 15 | Insert the catheter over the guide by advancing and then extend the guide until it exits through the distal lumen. |  |  |
| 16 | Advance the catheter to the correct distance (≤ 15 cm at right side, ≤ 18 cm at left side) |  |  |
| 17 | Remove the guide, checking that it is intact and occlude the distal lumen. |  |  |
| 18 | Confirms the correct intravenous situation by aspirating the blood and then flushing. |  |  |
| 19 | Confirms the correct position and absence of pneumothorax (usually radio). |  |  |

Strikethrough items are those that could not be observed in the study.

Removal of 11 items from the Checklist of Hartman et al (18):

*Item 2*: The students assume that the dummy is consenting and explain again the indication and the course of the procedure.

*Items 3, 4 and 7*: CVC is applied on a phantom dummy for the right internal jugular track. The students do not have the choice of the site or the positioning of the patient. Similarly, it is understood that the vein and artery do not present any anatomical difficulties or contraindications to puncture, so there is no identification with ultrasound prior to installation for the central line.

*Item 5*: Students ask for the necessary material but do not choose the type of material. The type of equipment depends on the availability of the simulation sessions.

*Item 13*: CVC lines are not infused at the end of the procedure.

*Items 19, 26, 27*: Students are asked not to make incisions in the skin of the phantom dummy in order not to damage the material prematurely due to the large number of punctures. Similarly, the fixation and dressing steps are discussed orally but not performed on the dummy.

*Items 28 and 30*: Students do not clear the material and do not confirm the use of the track, in order to have a faster turnover of sessions given the limited time and the large number of students.

## Global Rating Scale (GRS)

Translated from Hartman N et al, 2017. (18)

| Variable/  Rating | Global Rating Scale (GRS) | |  |  |  |
| --- | --- | --- | --- | --- | --- |
|  | 1 | 2 | 3 | 4 | 5 |
| Specific knowledge of the procedure | Significant lack of knowledge |  | Knows all the important steps of the procedure |  | Appears familiar with all the steps of the procedure |
| Knowledge of the equipment | Rather uses inappropriately |  | Knows the names of the equipment and uses it appropriately |  | Appears familiar with all instruments |
| Flow of the procedure | Frequent stops and seems insecure about the next step |  | Adequate planning and progression of the procedure |  | Easy and planned progression of the procedure |
| Time and movement | Numerous unnecessary movements |  | Time/movement  efficiency |  | Economy of movements |
| Getting to grips with the instruments | Repeated or inadequate attempts at movement by inappropriate grip |  | Competent but sometimes imperfect use of instruments |  | Smooth and precise movements |
| Two-handed  dexterity | One-handed operation, inadequate coordination |  | Uses both hands but not optimally |  | Easy to use with both hands |
| Sum of the scores obtained for each of the 6 spots (5-30) |  |  |  |  |  |
| Overall  Performance | Very low |  | Competent |  | Superior |
